# Body mass evolution as a driver of morphological and ecological diversity in terrestrial mammals

**DOI:** 10.1186/s12862-025-02393-9

**Published:** 2025-07-11

**Authors:** Priscila S. Rothier, Anthony Herrel, Roger B. J. Benson, Brandon P. Hedrick

**Affiliations:** 1 Department of Biomedical Sciences, College of Veterinary Medicine at, Cornell University, Ithaca, 14843 USA; 2 Département Adaptations du Vivant, Muséum National d’Histoire Naturelle, Paris, 75005 France; 3 Naturhistorisches Museum Bern, Bern, 3005 Switzerland; 4 Department of Biology, Evolutionary Morphology of Vertebrates, Ghent University, Ghent, B-9000 Belgium; 5 Department of Biology, University of Antwerp, Wilrijk, B-2610 Belgium; 6 Division of Paleontology, American Museum of Natural History, New York, 10024 USA

**Keywords:** Forelimb, Disparity, Locomotion, Mammalia, Size

## Abstract

Body mass plays a fundamental role in the macroevolutionary dynamics of morphological, ecological, and phylogenetic diversification. Given biomechanical principles, large body masses in terrestrial vertebrates may impose important constraints on the adaptative potential of skeletal morphology. This is especially true for the limbs, which are involved in both supporting and propelling the body during locomotion. We present a novel framework for evaluating how body mass structures patterns of morphological, ecological, and phylogenetic diversification using a dataset of forelimb traits for more than 600 terrestrial mammal species. We found that forelimb shape disparity increases with body mass for mammals generally as well as within mammalian subclades, suggesting that this trend is robust to phylogenetic scale. However, both phylogenetic and locomotor diversity (a proxy for ecological diversity) were high for all except the largest mammals and were not strongly associated with body mass. This suggests that small mammals are capable of speciating widely and evolving novel locomotor modes without requiring drastic changes to forelimb shape. However, as body mass increases, biomechanical constraints require substantial morphological changes to the forelimb to adapt to similar levels of locomotor mode disparity. We also show that different limb bone elements do not respond in the same way to increases in body mass when analyzed individually, perhaps due to differing developmental constraints. We provide new insights on how body mass structures macroevolutionary processes in mammals, and our approach can be generalized to examine this question for a variety of traits, ecological modes, and phylogenetic groups.

**Supplementary Information:**

The online version contains supplementary material available at 10.1186/s12862-025-02393-9.

## Introduction

Body mass is a fundamental driver of macroevolutionary change across Metazoa [1–8]. An animal’s mass profoundly impacts its ecological interactions [9, 10], physiology [11, 12], and biomechanics [13], thereby determining its place in the natural world [14]. As animals evolve larger body masses, they generally require more extensive home ranges and greater energetic resources to accommodate their absolute higher metabolic demands, meaning that the exploitation and diversification of new ecological niches between smaller and larger taxa may not be the same [15, 16]. As a result, body mass plays a central role in macroevolutionary dynamics and thus in a group’s tempo and mode of trait diversification [1, 7, 17–21].

The morphology of the tetrapod appendicular skeleton is primarily affected by body mass, given its functional role in terrestrial locomotion and support of the animal’s weight against gravity [22–26]. Larger animals generally have more robust, columnar limb bones, due to a hyperallometric relationship of body mass to limb cross-sectional ratios, whereby larger body masses require substantially higher cortical bone thickness to resist compressive forces [22, 27–30]. As body mass increases, animals commonly adjust their stride and speed [31], involving anatomical and physiological adaptations that reduce the metabolic cost of locomotion while simultaneously coping with the increased mechanical loads required in supporting and propelling greater body masses [13, 32]. Such adaptations are often observed in the structural configuration of the limb skeleton, including postural transitions from crouching in smaller vertebrates to more upright postures in large taxa, such as ungulates, elephants, and some dinosaurs [33–35]. The distal limb, in particular, exhibits numerous functional adaptations in conjunction with shifts in body mass, such as bony element fusion and/or reduction and proximal migration of muscle mass that lighten distal limbs and optimize weight support [36–40].

Small mammals may have a greater ability to diversify across various ecological niches due to reduced weight bearing constraints, implying that they may respond more flexibly to novel ecological opportunities than larger taxa [13, 41, 42]. Limited evidence for this hypothesis is available so far for rodents, which are an exceptionally diverse group and have repeatedly evolved diverse locomotor ecologies (e.g., semiaquatic, arboreal, semifossorial) without substantial changes to their proximal limb morphology [41]. More broadly, skeletal variation of small-sized mammals and birds is typically lower than in medium-sized species [20, 42, 43], which may reflect reduced skeletal integration and mechanical adaptation associated with weight support [20, 41, 44]. Given that increased integration has been hypothesized to impact evolvability [45–47], the relationship between limb integration and body mass could act as a constraint, limiting the potential for morphological and ecological diversification within large body mass lineages.

The role of body mass on generating phenotypic diversity had been long proposed as foundational in macroevolution [16]. Despite this, few studies have examined how body mass structures macroevolution across many species and groups (but see [42], which compared morphological diversity in two size categories of mammals – small: 0.005–0.47 kg versus medium: 0.47–12.8 kg). Instead, the majority of studies assessing body mass focus on allometric scaling relationships [24, 27, 28, 48–51], rates of morphological diversification [1, 8, 17], or body mass’ effect on other traits such as diet and geographic distribution [10, 52–54]. While valuable, these approaches do not directly inform the question of how body mass variation impacts the macroevolutionary potential to generate morphological and ecological variability.

**Fig. 1:**
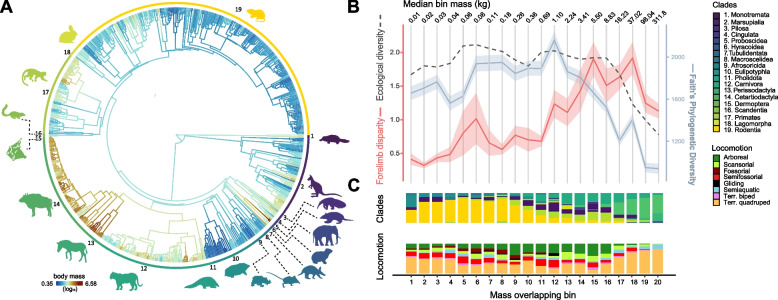
Evolution of body mass in terrestrial mammals and its relationship with morphological disparity, locomotor mode, and phylogenetic diversity. **A** Ancestral reconstruction of body mass (log_10_ in grams) for the terrestrial mammals analyzed. **B** Association between mass (“mass overlapping bin”, with bin median values in kg indicated on the top) on forelimb shape disparity (red), phylogenetic diversity (blue), and locomotor mode ecological diversity (dotted black), **C** indicating the proportion of clades and locomotor modes represented in each overlapping bin. Disparity and ecological diversity values are multiplied by 10 to facilitate interpretation

Living terrestrial mammals exhibit variation in body mass across six orders of magnitude, from the diminutive 2 g Etruscan shrew to 6000 kg African elephants, the largest range among living land vertebrates (Fig. 1A [55, 56]) – without mentioning extinct groups such as giant rhinos, that have reached colossal body masses of 10–20 tonnes [57]. Their size diversity, along with their ecological and locomotor diversity, makes terrestrial mammals an ideal group for understanding how body mass structures the diversification of both appendicular skeletal morphologies and locomotor ecologies [39, 58, 59]. We present a novel framework for addressing how body mass impacts macroevolutionary patterns of ecomorphological diversification of the mammalian forelimb. Our approach accounts for the continuous nature of body mass variation by sampling species into moving windows, or partially overlapping body mass bins, to determine how increases in body mass affect forelimb shape disparity, locomotor mode diversity, and phylogenetic diversity. While the hind limb in terrestrial animals is fundamental in supporting body mass in terrestrial mammals, we chose to focus on the forelimb because this structure is typically more variable than the hind limb, possibly due to its greater range of functional roles beyond supporting the animal’s weight [39, 60]. Additionally, the forelimb’s widespread presence across all mammalian species facilitates future comparisons of our approach with aquatic taxa that have undergone total hind limb loss, such as cetaceans and sirenians. To characterize forelimb shape, we collected forelimb morphometric data from 666 species of mammals, spanning 95% of extant family-level diversity of terrestrial lineages. We predict that forelimb shape disparity will increase with body mass given that increasing weight imposes greater morphological constraints on biomechanics and thus requires more distinct limb morphologies for divergent locomotor modes. We further anticipate that smaller taxa will be more phylogenetically and ecologically diverse than larger taxa due to greater biomechanical adaptations required at high body mass. We expect that these patterns will be stronger in the distal limb than the proximal limb. Finally, we predict that the patterns that we see across mammals will also be evident in mammalian subclades. Our study aims to understand how body mass, a major constraint on morphological macroevolution across animals, simultaneously drives the evolution of morphology, ecology, and phylogenetic diversity in mammals. Our approach additionally provides a framework for investigating how body mass has affected macroevolutionary patterns in other groups of animals.

## Material and methods

### Morphological and ecological data

Data were collected from 666 species of terrestrial mammals (Fig. 1A) using a subset of the data published by [59] supplemented with additional nine species of Rodentia, Afrosoricida, and Eulipothyphla, to increase sample sizes for the smallest mammals (703 specimens analyzed in total). Sampling consisted of one to three individuals per species, encompassing all living orders of terrestrial mammals (60% and 90% of extant genus and family diversity of terrestrial mammals respectively [61, 62]. Fully aquatic species (cetaceans, pinnipeds, and sirenians) and flying species (bats) were excluded since their locomotor regimes impose very specific physical constraints on mass and limb shape leading them to differ widely from terrestrial groups [59]. Marsupial moles (Notoryctemorphia) were also excluded due to fused phalanges in their third digit that prevented the acquisition of some morphological measurements. Sex data were commonly unavailable for many specimens included in the dataset and thus the effects of sexual dimorphism (whether in terms of morphology or body mass) are not accounted for in our analyses.

To characterize forelimb morphology, three linear distances (length, proximal width, distal width) were measured from the humerus, radius, third metacarpal, and proximal phalanx of digit III, totaling 12 linear measurements per animal. Data acquisition was performed either digitally from 3D digital models (424 specimens) or manually using calipers (279 specimens) (detailed in Dataset S1). All individuals were measured twice by the same researcher, and average values for each measurement were used in downstream analyses. Error was generally below 1.5% regardless of an individual’s size or measurement method used, indicating reliability and repeatability of the methods employed [58, 59]. Measurements from multiple individuals of the same species were averaged, producing species mean values. Body mass estimates per species were compiled from the PanTHERIA database [63] and primary literature (see Dataset S1). Measurements and body masses were log_10_-transformed prior to analyses. To obtain a proxy of body size, we also calculated the geometric means of all measurements used, in addition to the midshaft height and width of each bone (see [58, 59].

Species were classified into locomotor modes categorically according to their predominant form of locomotion using ecological descriptions provided by Nowak [64] and complemented with additional sources (e.g., [41, 59, 65]; see Dataset S1 for full list). Taxa were assigned to eight locomotor modes: (1) arboreal (primarily uses arboreal surfaces for foraging, resting, and traveling, although capable of moving on the ground); (2) fossorial (constructs their own borrows, primarily underground for traveling, foraging, and resting); (3) gliding (capable of moving through air using a patagium); (4) scansorial (spends time both on the ground and in trees for foraging, escaping, or resting); (5) semi-aquatic (efficiently moves both in water and on the ground, but highly dependent on aquatic environments for foraging); (6) semi-fossorial (makes considerable use of subterranean and ground substrates and may be capable of digging for foraging and shelter, but does not construct complex burrows); (7) terrestrial biped (bipedal locomotion on the ground by hopping, saltation, or walking); (8) terrestrial quadruped (quadrupedal locomotion on the ground, such as in grass, sand, rock, leaf litter, etc.).

To account for phylogenetic relatedness of the taxa and phylogenetic uncertainty [66–68], we randomly assembled 100 posterior trees from Upham et al. [69] pruned to include all of our taxa. All used trees are available with the supplementary codes (https://github.com/psrothier/mammal_bodymass). To address differences in size across our taxa, we then generated residuals of forelimb measurements and corresponding geometric means by fitting phylogenetic generalized least-squares regressions (PGLS [68]) under a Brownian Motion model of evolution. The PGLS was conducted with the mvMORPH R package v. 1.1.9 for all 100 trees [70]. This resulted in 100 shape residuals datasets for each bone for all taxa.

### Comparisons of body mass, forelimb shape disparity, phylogenetic diversity, and ecological diversity across mammals

To examine how disparity of the limb skeleton varies with body mass, we utilized modified moving windows approach, or partially overlapping bins, a method usually used for smoothing historical data and used here for grouping taxa based on body mass [20, 71]. We adopted this method to address the limitations of artificial categorizations of species based on average body mass. Given that individual body mass can deviate from the average, this approach allows for variation in bin assignments by creating an overlap between bins rather than placing species into fixed categories.

All species were initially split into 20 fixed bins of increasing body mass (each bin contained 33–34 taxa). From these bins, 20 moving windows were then generated such that each partially overlapping bin contained 44 species. For the first overlapping bin (which lacks a previous adjacent bin), the 10 lightest species from fixed bin 2 were included, and for the final overlapping bin, the 10 heaviest individuals from fixed bin 19 were included. For all intermediate overlapping bins, five to six species from the preceding (heaviest species) and subsequent (lightest species) fixed bins were included. The number of bins and the degree of overlap between them were evaluated through a sensitivity analysis. This involved splitting the species based on their orders of magnitude of body mass, and then replicating the analyses described below using various binning strategies (10, 15, 20, 25, and 30 fixed bins; Fig. S1-2). We highlight that the chosen binning method may vary according to the researcher’s goal. Since the main results remain consistent across all binning methods (see Results and Fig. S3-4), we determined that 20 bins overlapping between 15–18% with their adjacent bins (but first and last bins having 29% of species of the adjacent groups) illustrate an ideal balance between reducing noise and preserving meaningful differentiation between categories (Fig. S1-2). For future studies, it is encouraged to conduct similar sensitivity analyses and consider similar factors to determine an appropriate binning strategy, ensuring that such approach is repeatable and justifiable.

Following bin construction, we calculated morphological disparity, phylogenetic diversity, and locomotor mode ecological diversity for each overlapping bin. To calculate changes in morphological disparity, we split the size residuals into subsets defined by the overlapping bins in dispRity v. 1.8 [72]. We then ran 100 permutations across each overlapping bin and calculated the disparity with the permutated data as the sum of variances [73]. This procedure was repeated with the shape residuals generated from all 100 posterior trees. To assess changes in phylogenetic diversity, we calculated Faith’s phylogenetic diversity, which corresponds to the sum of the branch lengths of a tree [74], using EcoPhyloMapper v. 1.1.1 [75]. Finally, ecological diversity was estimated using Gower distances between locomotor ecologies. We generated a single distance value representing the mean locomotor mode ecological diversity within each overlapping bin using cluster v. 2.1.6 [76].

To investigate the relationships between body mass, forelimb morphological disparity, phylogenetic diversity, and locomotor mode ecological diversity, we performed a non-parametric test using Kendall’s τ rank correlation coefficient. For this analysis, we retained the mean Gower distances of each overlapping bin and calculated the mean overlapping bin values for forelimb shape disparity and Faith’s phylogenetic diversity across all 100 posterior trees. We used median body mass values rather than means within each overlapping bin since it is resistant to outlier species that are extremely heavy or extremely light. We repeated the correlation tests by dividing the dataset into the ten smallest (from 2.3 to 558 g) and ten largest (from 445 g to 3,800 kg) overlapping bins to evaluate whether overall results were similar for smaller and larger taxa considered separately. We replicated the correlation analyses also with the different binning strategies to verify how much the chosen method impacts on the patterns detected. We also ran a conventional Principal Component Analysis using the forelimb data to visualize the distribution of species and locomotor modes from each bin in the overall mammalian morphospace.

Next, we examined variation in the disparity of individual bones to examine how patterns may change across a proximodistal gradient within the forelimb. Size residuals from the humerus, radius, metacarpal III, and the proximal phalanx of digit III were separately calculated using the geometric mean of the entire forelimb in a PGLS for all 100 trees (as above for the full dataset). Both binning methods and disparity calculations across bins for individual bones were done identically as for the full dataset ensuring results were comparable, also verifying whether the binning method impacted the resulting patterns. Finally, relationships between bone disparity, body mass, and phylogenetic and locomotor mode ecological diversity were compared using Kendall’s τ rank correlation coefficient, as above.

To verify whether the macroevolutionary patterns detected for Mammalia are also present at a subclade level, we replicated this procedure by pruning each full tree into trees that only included subclades for which there were greater than 35 sampled species: Marsupialia (63 species), Rodentia (254 species), Primates (66 species), Cetartiodactyla (86 species), Eulipothyphla (39 species), and Carnivora (89 species). Given differences in sample sizes across subclades, we used different numbers of fixed bins for each group: Marsupialia (6 bins, 10–11 species per bin), Rodentia (10 bins, 25–26 species per bin), Primates (6 bins, 11 species per bin), Cetartiodactyla (8 bins, 10–11 species per bin), Eulipothyphla (5 bins, 7–8 species per bin), and Carnivora (8 bins, 11–12 species per bin). Overlapping bins were later assigned for each subclade, each containing 16 species, except for Rodentia (*n* = 32) and Eulipothyphla (*n* = 12). We have not performed a correlation test between body mass and forelimb shape disparity across mammalian subclades because the number of overlapping bins designed for each subclade was too small for robust estimates.

## Results

The overall patterns of correlation detected between limb shape disparity, Faith’s phylogenetic diversity, and Gower’s distance, were consistent across all binning methods (Fig. S3-S4), reinforcing the reliability of the binning method chosen. Significance slightly varied by approach, so we opted to solely present the results that have repeatedly exhibited significant Kendall’s τ rank larger than 0.4. We detected a generally increasing trend of forelimb shape disparity along body mass bins (Fig. 1B) revealing a significant and positive association between forelimb disparity and body mass (τ = 0.61, *p* < 0.05; Fig. S3). The lowest disparity values are observed across the first four overlapping bins, in which mammals range from 2.3–53.3 g and include small rodents, eulipotyphlans, marsupials, tenrecs, and elephant shrews (Fig. 1B-C, Fig. S5-6). Overlapping bins 5 and 6 have high disparity compared to adjacent bins and represent the body mass ranges that encompass most talpid species, in addition to many rodents and afrosoricids (Fig. 1B-C, Fig. S5-6). This increasing trend in forelimb shape disparity continues until bin 18 (median mass 37 kg), where maximum forelimb shape disparity within a mass range is observed. This bin comprises a wide diversity of ungulates, carnivores, marsupials, xenarthrans and primates, including *Homo sapiens*. As mammals reach masses larger than 52 kg (bins 19 and 20), limb shape disparity exhibits a drastic decrease compared to previous mass ranges.

The relationship between forelimb disparity and body mass is weaker when analyzing just smaller-bodied mammals (bins 1–10) or larger-bodied mammals (bins 11–20), than when analyzing across all body mass bins. A significant, but low correlation is detected within the smallest taxa (τ = 0.51, *p* = 0.046). Across the largest taxa, we did not detect a significant association between mass and forelimb shape disparity (τ = 0.33, *p* = 0.22) (Fig. S3).

Faith’s phylogenetic diversity is relatively constant until overlapping bin 12 (median mass 1.1 kg, ranging from 811 g – 1.6 kg), where these values peak and are followed by a drastic decrease. Phylogenetic diversity is not correlated with forelimb disparity but is negatively correlated with body mass at the largest size bins (bins 11–20; τ = −0.91, *p* < 0.05; Fig. S3). As phylogenetic diversity decreases across larger mass bins, the proportion of ungulates increases (Fig. 1C).

Locomotor mode ecological diversity and limb shape disparity are only significantly associated in small mammals (bins 1–10) such that skeletal diversity increases with locomotor mode ecological variation (τ = 0.6, *p* < 0.05). Locomotor mode ecological diversity is correlated with Faith’s phylogenetic diversity when all size bins are considered (τ = 0.56, i < 0.05), but is particularly strong for larger bins (τ = 0.87, *p* < 0.05) (Fig. S3-4), indicating a strong evolutionary structure underlying the diversity of locomotor modes. Locomotor mode ecological diversity is relatively high and stable among smaller body mass groups but rapidly decreases at higher body masses (specifically at size bin 17, median mass of 37 kg), exhibiting a strong and negative correlation (τ = −0.87, *p* < 0.05). While all locomotor categories are well represented across mammals weighing up to 500 g (bin 11), the frequency of highly specialized locomotor modes (e.g., arboreality, fossoriality, and gliding) either decreases in prevalence or completely disappears at larger body masses. This results in mass bins that are increasingly represented by terrestrial taxa.

**Fig. 2:**
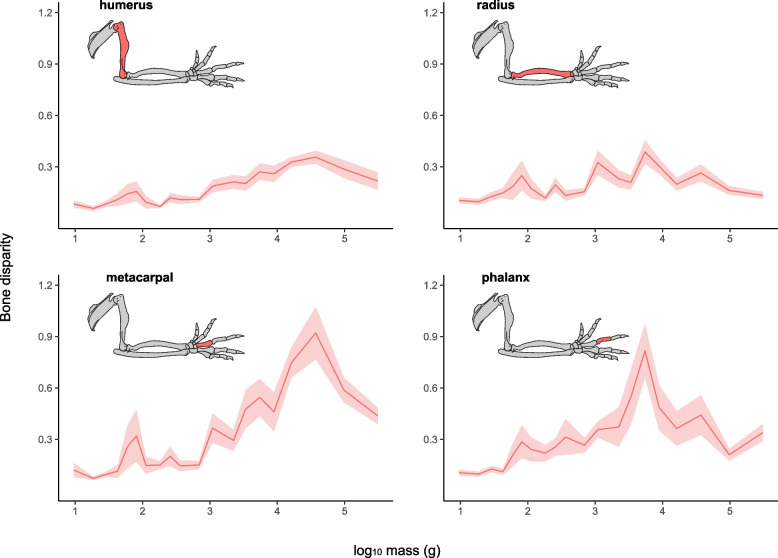
Bone shape disparity. Phenotypic disparity of each bone examined (humerus, radius, metacarpal and phalanx) across overlapping mass bins. The ‘x’ axis indicates the average mass value of each bin in grams (log_10_). Disparity values are multiplied by 10 to facilitate interpretation

Different binning methods also revealed consistent correlation patterns between disparity, Gower’s distance, and Faith’s phylogenetic diversity when the individual bones were examined (Fig. S7-15). The significant, positive association between forelimb shape disparity and body mass was recovered for each individual forelimb bone (Fig. 2) (Fig. S7). However, the degree of correlation differed across forelimb elements. While the humerus has the strongest correlation between body mass and shape disparity (τ = 0.72, *p* < 0.05), it has low overall disparity values, and little difference, or amplitude, between the lowest (bin 2) and largest (bin 18) mean disparity across size bins (difference = 0.3). Metacarpal III has the second strongest correlation between disparity and mass (τ = 0.68, *p* < 0.05) and exhibits the greatest overall disparity and amplitude (difference = 0.9) between the lowest (bin 2) and greatest (bin 18) mean values. Across forelimb elements, metacarpal III most closely resembles trends for overall forelimb disparity structure. The proximal phalanx of digit III also presents a strong correlation with body mass (τ = 0.60, *p* < 0.05), but shape disparity peaks at a more intermediate body mass (bin 15, mean mass 5.5 kg, a bin with a particularly high proportion of xenarthrans). The radius has the lowest correlation between shape disparity and body mass (τ = 0.38, *p* < 0.05; with most binning methods not recovering such a significant correlation – Fig. S13) and the lowest amplitude (difference = 0.28). Except for the proximal phalanx of digit III, all bones exhibit a decrease in disparity in the last two size bins, similar to the pattern detected for forelimb shape disparity as a whole.

**Fig. 3:**
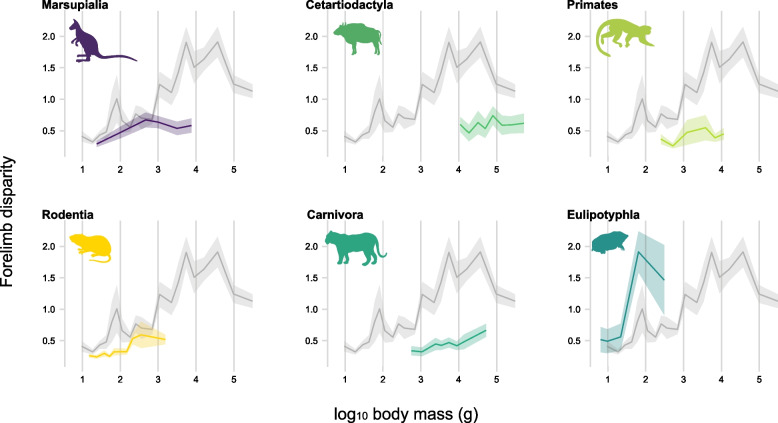
Mammalian subclade disparity. Forelimb shape disparity within six major mammalian subclades (Marsupialia, Cetartiodactyla, Primates, Rodentia, Carnivora) compared to the overall disparity detected among all terrestrial mammals (in gray). The ‘x’ axis indicates the average mass (log_10_ grams) of each overlapping bin. Disparity values are multiplied by 10 to facilitate interpretation

Mammalian subclades show similar patterns to the overall mammalian pattern (Fig. 3). Such pattern of increased forelimb disparity across larger species is notably observed in carnivores, rodents and eulipotyphlans, and to a lesser extent in marsupials (Fig. 3). Cetartiodactyla and Primates do not exhibit a clear pattern of increasing forelimb disparity in larger bins. Notably, eulipotyphlans weighing more than 60 g exhibit extremely high forelimb shape disparity. This mass range includes taxa with fundamentally different body shapes and sizes, including *Solenodon*, moles, and hedgehogs. This peak in eulipotyphlan disparity matches that observed for mass bins 5 and 6 across all terrestrial mammals demonstrating that it is not only high in eulipotyphlans, but that eulipotyphlans have high forelimb shape disparity relative to mammals as a whole (Fig. 3).

## Discussion

Body mass has a pervasive influence on nearly every aspect of a species’ ecology, life history, and evolution. We show that the disparity of forelimb morphologies increases with body mass in mammals, whereas the diversity of locomotor modes decreases with increasing body size. This provides strong evidence that body mass variation structures patterns of phenotypic evolution across mammals, and most likely across vertebrates, more generally. Although small and medium sized mammals exhibit less overall forelimb disparity than large mammals, they have greater locomotor diversity. Conversely, the largest species are confined to a few mammalian lineages with low locomotor diversity. We additionally found that forelimb shape disparity increases with body mass when considering each forelimb element individually. However, this occurs at different magnitudes, suggesting possible proximodistal gradients in forelimb disparity patterns and differential responses to body mass evolution. Finally, the trends for increased forelimb shape disparity were also detected within major mammalian subclades, indicating that these general patterns occur at multiple phylogenetic levels.

### Biomechanical limitations of forelimb shape disparity

We show that mammal species weighing less than 50 g predominantly display less specialized, more generalist locomotor modes, such as terrestrial and semi-fossorial habits. At these smallest sizes (< 50 g), animals also move at slower speeds [77], limiting their ability to effectively explore their surrounding three-dimensional environment. This limitation likely confines these mammals into terricolous modes, often seeking refuge in burrows [64]. As mammals grow larger than 50 g, they are more likely to employ multiple locomotor modes for navigating heterogenous terrain and micro-environments. Species ranging from 50 g to 13 kg (bins 5–16) exhibit higher proportions of specialized locomotor ecologies (e.g., gliding, arboreal, fossorial, semi-aquatic). Many mammal species ranging from 50 to 500 g are capable of utilizing a range of locomotor modes (e.g., scansorial, semi-fossorial, and semi-aquatic behaviors), such as the South American water rat *Nectomys squamipes*, which are considered semi-aquatic rodents, but are also agile climbers and sprinters [78, 79].

At masses larger than 500 g, mammals are both more anatomically and functionally differentiated [42] such that they have increased locomotor specialization and phenotypic disparity. At these sizes, body mass imposes greater constraints on locomotor biomechanics than in smaller species, making it harder for these taxa to efficiently move across a wide range of heterogenous substrates. After reaching body masses greater than 50 kg, our data suggest that these constraints become more pronounced, resulting in a decrease in forelimb shape disparity, locomotor mode ecological disparity, and phylogenetic disparity (Fig. 1B). A similar pattern is also observed within primates, in which locomotor diversity tends to decrease with mass, as large apes lose arboreal capabilities due to weight constraints [80].

Mammals typically cope with increased mechanical stress due to higher body mass by evolving more robust long bones and an upright posture which provide increased biomechanical advantage for the limb muscles and joints over gravitational constraints [30, 81, 82]. Likewise, animal speed initially increases with mass, as larger animals typically have longer limbs capable of producing greater stride lengths [81]. Limb straightening, however, reaches a plateau around 300 kg, a mass range corresponding to approximately the size of a horse [83]. This reduces biomechanical advantage, contributing to the adoption of slower speeds that reduce mechanical stress [77]. An inverse relationship between size and speed has been observed in bovids and large carnivores [84] and has been detected for other large tetrapods, such as varanid lizards [81]. We hypothesize that the pattern of decreasing forelimb shape disparity in large mass bins is also derived from the extreme biomechanical constraints that gigantic terrestrial taxa are subjected to, which compared to medium-sized taxa, leads to a reduced set of possible bone shapes. Such constraints are likely also reflected in the low locomotor disparity displayed by these largest taxa, that are more predominantly confined to terrestrial and cursorial modes [34]. Indeed, there are no arboreal, fossorial, or gliding taxa the size of horses or elephants.

It is important to highlight that phylogenetic diversity is also a central factor influencing the low forelimb shape disparity observed in large mammals. Low phylogenetic diversity for the largest bins reveals that, at least among extant taxa, the evolution of gigantic forms was achieved by few mammalian lineages. In general, smaller mammal species are more common than larger species with ~ 70% of living terrestrial mammals (excluding bats) weighing less than one kilogram [63]. Because strong phylogenetic signal underlies the evolution of large body masses [85, 86], reduced shape variability is expected to be detected for these groups due to their recently shared ancestry. On the other hand, our approach of including only extant taxa does not fully capture the diversity of body sizes that have evolved throughout the mammalian tree of life. Among recently extinct species in the Anthropocene, there were additional taxa that reached these largest body sizes that are in lineages for which large taxa still exist (e.g., mammoths, mastodons, wooly rhinoceros), but also lineages that have lost their largest species (e.g., giant ground sloths, glyptodontids, notungulates, marsupials, hyracoideans [83, 87]). The rapidly increasing extinction rates experienced by these large mammals [88, 89] have undoubtedly impacted both the results of our work and our broader understanding of biodiversity and evolution. Although including these taxa would increase the phylogenetic diversity over the largest mass bins, it is unclear that it would also strongly increase forelimb shape disparity. Nevertheless, including fossils may improve the resolution of the disparity trends and provide further insights on the patterns of limb transformation with increasing body mass.

### Functional and developmental constraints on within limb variation

Although all forelimb bones collectively play a structural role in supporting body weight in terrestrial mammals, body mass impacts the shape disparity of forelimb elements in different ways, leading to potentially different trajectories across the forelimb. The humerus and radius exhibit overall low shape disparity, while the autopodial bone shapes, particularly that of the metacarpal, exhibit a strong relationship of disparity scaling with increasing body mass. These findings align with previous research documenting uneven evolutionary diversity along the proximo-distal axis of tetrapod forelimb elements [58, 90]. Autopodial bones, which are the last elements to condense during limb ontogenesis, are less developmentally constrained [91] and more morphologically labile in their evolutionary responses [58]. The hands and feet directly interact with substrates, absorbing impact shocks as size increases [83]. As body mass grows larger and body posture becomes increasingly upright, several taxa display autopodial adaptations that attenuate mechanical stress, such foot pads in rhinos and elephants [92–94], the development of a sesamoid into a “sixth” digit in elephants [95], digit loss in ungulates, and metacarpal fusion in artiodactyls and enlargement in horses [36, 37, 96]. Both developmental and functional diversity may in turn affect the relationship between body size and autopodial variability more directly across mammals.

Although the humerus shape is less variable than the autopodium, its disparity does depend on body mass, given that humeral shape and position are crucial in determining body posture. The humerus anchors the limbs to the pectoral girdle and assumes a more horizontal orientation in crouching postures, while it is positioned vertically in upright postures [97]. Consequently, humeral morphology plays an important role in locomotor kinematics [98, 99]. Many small to medium-sized crouching mammals, such as many rodents and carnivoran species, display weak allometric trends in the humerus [41, 49] and locomotor biomechanics of the forelimb are conserved across different lineages [99]. Both developmental constraints and biomechanical homogeneity in small and medium-sized taxa might explain the overall reduced disparity in the humerus compared to other bones. However, as mammals become larger and assume a more upright posture, the humerus exhibits a stronger allometric trend described by increasingly robust and sturdy shapes [27, 28, 36]. As a result, body mass, and consequently body posture, are strong predictors of humeral variability across Mammalia as a whole.

Morphological aspects of the mammalian radii, such as curvature, exhibits unique scaling patterns compared to other long bones. Curvature variation is independent of size among small- and medium-sized taxa, but the radius becomes straighter in animals heavier than 50 kg [100]. Low radial shape disparity among small-sized mammals implies that postural changes, and not anatomical modifications, are used for reducing mechanical stress during locomotion. Furthermore, the zeugopodium exhibits the most conserved relative proportions within the forelimb across Tetrapoda, most often encompassing approximately one-third of total forelimb length [101]. The stylopodium and the autopodium, on the other hand, vary in a trade-off pattern of elongation and reduction, encompassing the remaining two-thirds of limb proportions [101]. Both morphological and genetic data suggest that developmental constraints established during early ontogeny determine limb proportions through an activation–inhibition pathway [101], thereby limiting the morphological variation in zeugopod’s relative size [102]. These developmental features, coupled with a more plastic biomechanical role during weight support, may explain the reduced relationship between body mass and shape disparity of the radius compared to the humerus and the autopodium. Additionally, while our length metrics capture forelimb shape, they do not capture curvature. Methods such as three-dimensional geometric morphometrics, which capture shape more holistically, may present a more nuanced view of how radial shape disparity relates to body mass.

It is important to note that we chose an approach of using geometric means based on the forelimb measurements to conduct size correction. While this offers valuable insights into the variation within limb segment proportions, it may obscure significant sources of variation in overall limb proportion relative to body size. By incorporating metrics beyond the forelimb itself as a proxy of body size, such as skull traits, vertebral column length, etc. [50, 103], future works may be able to determine whether overall limb lengths are more variable across smaller, medium or lager species.

### Functional specializations unrelated to weight support

Forelimb and individual element shape disparities peak within a mass range between 49 and 100 g (overlapping bins 5 and 6), a size range that includes various talpid moles (Figs. 1B, 2). The impact of talpids on forelimb shape variation is most pronounced within the Eulipotyphla-only analysis (Fig. 3). These animals exhibit several ecological specializations, with fossorial animals in particular displaying highly specialized anatomical modifications in their forelimbs, such as extremely enlarged long bones relative to body size with large muscle attachment sites, additional sesamoids in the hand, and a secondarily derived sprawling posture [44, 104, 105]. Highly modified limb morphologies involved with digging have also evolved in other fossorial mammals, including many rodents, xenarthrans, marsupials, and golden moles. Due to phalangeal fusion in digit three, we did not include golden moles in our dataset, so their shape impacts are not considered here. However, they are similar to talpids in body mass, with body masses ranging between 30–100 g [106, 107]. The giant golden mole has the largest body mass among golden moles (~ 450 g), which is similar in size to the Russian desman, the largest talpid species [108]. As a result, their inclusion would likely increase the amplitude of the forelimb shape disparity peak seen for bins 5–6. These extreme adaptations are shaped by the need to traverse granular, dense media [109, 110]. The evolution of fossoriality, therefore, drives morphological diversity and the expansion of the limb morphospace in mammals, even in small taxa [41, 65, 111, 112]. This suggests that ecological specialization, rather than body mass, is a stronger predictor of phenotypic variability among these species.

Similar to fossoriality, aerial and aquatic locomotion are also governed by fluid dynamic principles [113] and like subterranean lifestyles, these modes contribute to the diversification of extreme morphologies and the expansion of limb morphospace [59, 112, 114, 115]. These clades, however, were not included in the present study because body mass and locomotion are subject to different physical constraints compared with terrestrial species [113]. During mammalian evolution, bats achieved powered flight, while other lineages such as pinnipeds, cetaceans, sirenians, and sea otters, developed primarily aquatic lifestyles, with both transitions involving numerous adaptations to both body mass and forelimb morphology [116–120]. While bats are confined to lighter masses and a relatively low diversity of forelimb morphologies, whales evolved a wide range of flipper anatomies and body sizes, including extreme gigantism [59, 121–124].

## Conclusion

Using a comprehensive large dataset of forelimb measurements spanning across 95% of terrestrial mammal families, we provide new insights on how body mass, a fundamental trait for an animal’s biology, drives ecomorphological diversity using a novel, adaptable approach for comparing body mass, shape disparity, ecological locomotor mode, and phylogenetic diversity. We demonstrate that although small mammals have relatively limited forelimb shape disparity, they have high locomotor diversity suggesting that they can shift locomotor modes without drastic morphological changes. Forelimb shape disparity tends to increase with body mass in mammals, but both diminutive and gigantic mammals exhibit more constrained phenotypic and ecological variation, likely due to biomechanical limitations related to weight support. This positive correlation between body mass and forelimb disparity is widespread across mammalian subclades. However, different forelimb elements do not respond in the same way to increases in body mass, indicating that while body mass is a significant driver of phenotypic variation, it does not affect all traits equally. In size categories that are predominantly fossorial, for instance, function predicts diversity more strongly than size. We hope our approach and these findings in mammal forelimbs will inspire new research on the central role of body mass in generating phenotypic diversity across different traits and clades across animals.

## Supplementary Information

Supplementary Material 1.

## Data Availability

Codes and data are available on GitHub: https://github.com/psrothier/mammal_bodymass.
