# Supplementary material for: Body mass evolution as a driver of morphological and ecological diversity in terrestrial mammals
Source: BMC Ecol Evol. 2025 Jul 11;25:69. doi: 10.1186/s12862-025-02393-9 (PMC12247252; doi:10.1186/s12862-025-02393-9)
Supplement: Supplementary file 1 — Supplementary Material 1. [file 12862_2025_2393_MOESM1_ESM.docx]

SUPPORTING INFORMATION

**Body mass evolution as a driver of morphological and ecological diversity in terrestrial mammals**

Priscila S. Rothier, Anthony Herrel, Roger B. J. Benson, Brandon P. Hedrick

**Dataset S1. List of specimens analyzed.** This dataset details the specimen’s museum ID, taxonomic and ecological classifications (with corresponding references), method of data acquisition, MorphoSource ID and corresponding supporting grant (when applicable), species body mass (referenced – otherwise, extracted from PanTheria), rolling bin classification, and raw morphological values.

**
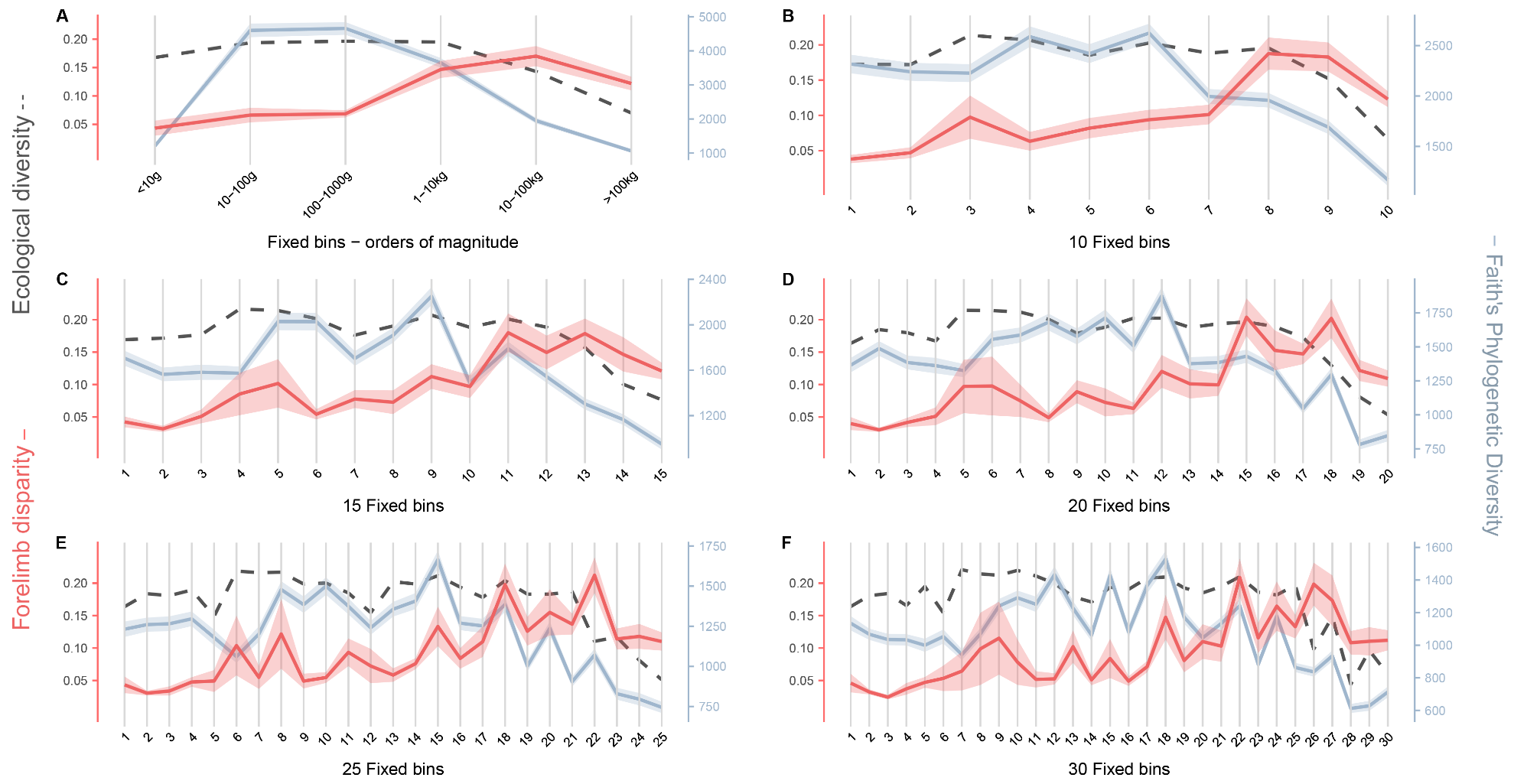
**

**Figure S1. Comparison of fixed binning methods on forelimb disparity, ecological diversity and Faith’s phylogenetic diversity.** A) Species classified based on orders of magnitude; B) 10 fixed bins (66/67 species); C) 15 fixed bins (44/45 species per bin); D) 20 fixed bins (33/34 species per bin) *; E) 25 fixed bins (26/27 species per bin); F) 30 fixed bins (22/23 species per bin). * Binning number chosen to conduct downstream analyses.


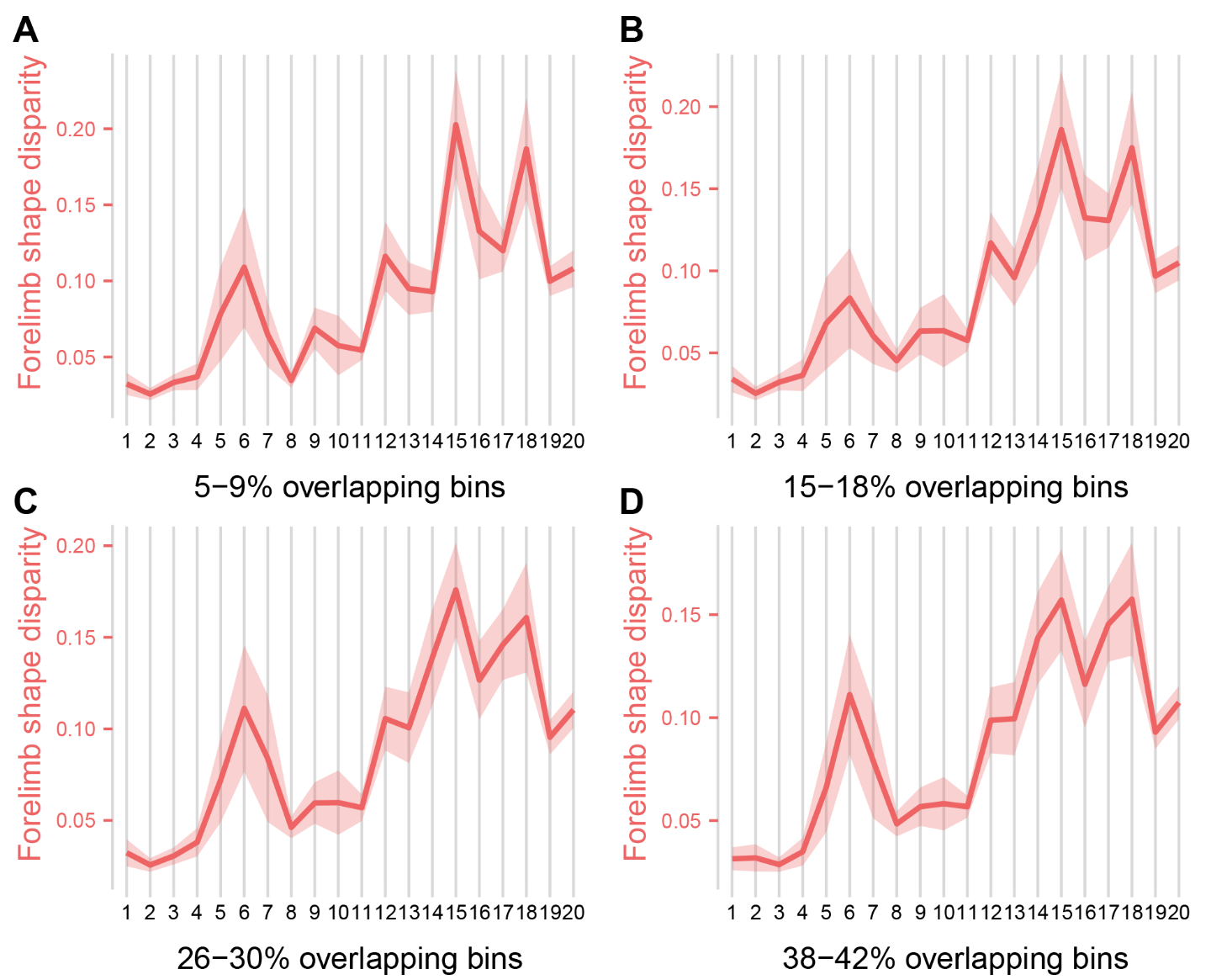


**Figure S2.** **Comparison of overlapping degrees on forelimb disparity.** A) Overlap of 5-9% between adjacent intermediate bins, and 12% in the edges; B) Overlap of 15-18% between adjacent intermediate bins, and 29% in the edges *; C) Overlap of 26-30% between adjacent intermediate bins, and 55% in the edges; D) Overlap of 38-42% between adjacent intermediate bins, and 76% in the edges. * Overlapping range chosen to conduct downstream analyses.


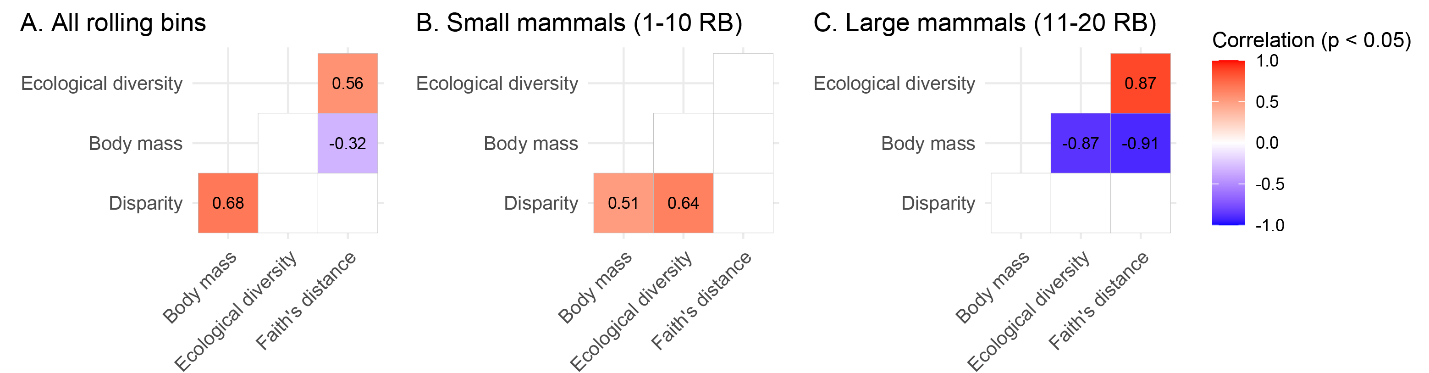


**Figure S3. Correlation between forelimb shape disparity, body mass, ecological diversity and Faith’s distance**. The heatmaps illustrate the results of significant values obtained from a Kendall correlation test. A) Correlation across all mammals (all rolling mass bins); B) Correlation within the smallest mammals (mass bins 1–10); C) Correlation within the largest mammals (mass bins 11–20).


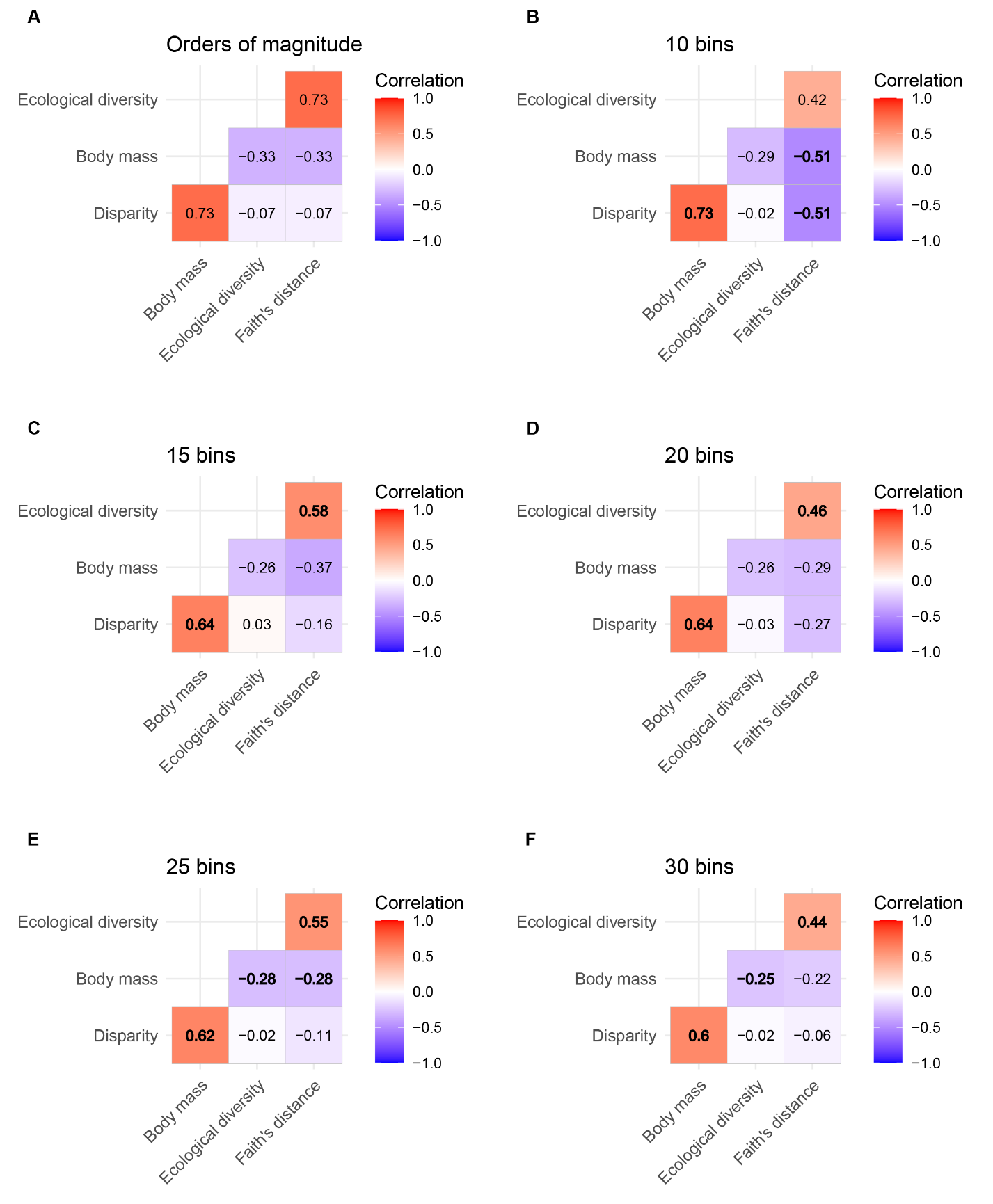


**Figure S4.** **Correlation between forelimb shape disparity, body mass, ecological diversity and Faith’s distance with different fixed binning methods.** The heatmaps indicate the degree of correlation obtained from a Kendall correlation test. Values in bold are significant. A) Binning based on orders of magnitude; B) 10 fixed bins; C) 15 fixed bins; D) 20 fixed bins; E) 25 fixed bins; F) 30 fixed bins.


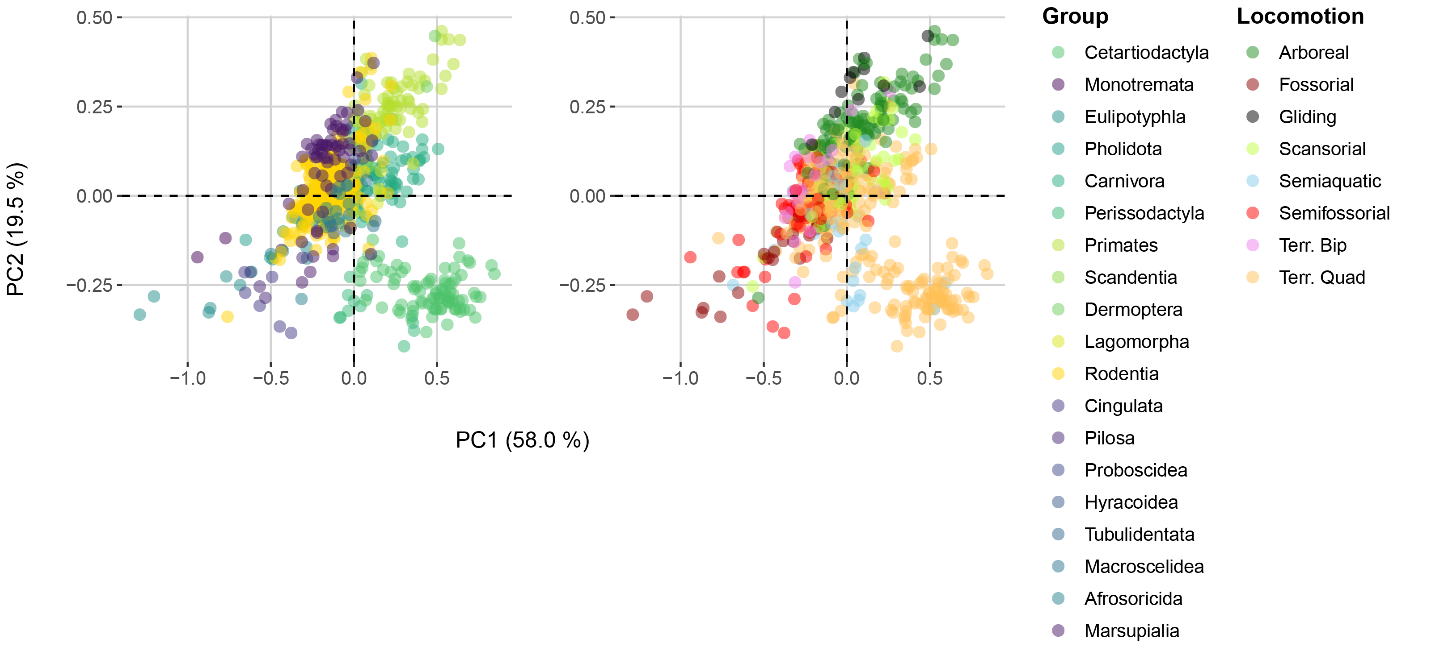


**Figure S5. Forelimb morphospace of terrestrial mammals.** Two first principal components describing the forelimb shape of terrestrial mammals, indicating the major clade (left panel) and locomotor mode (right panel) of each species.


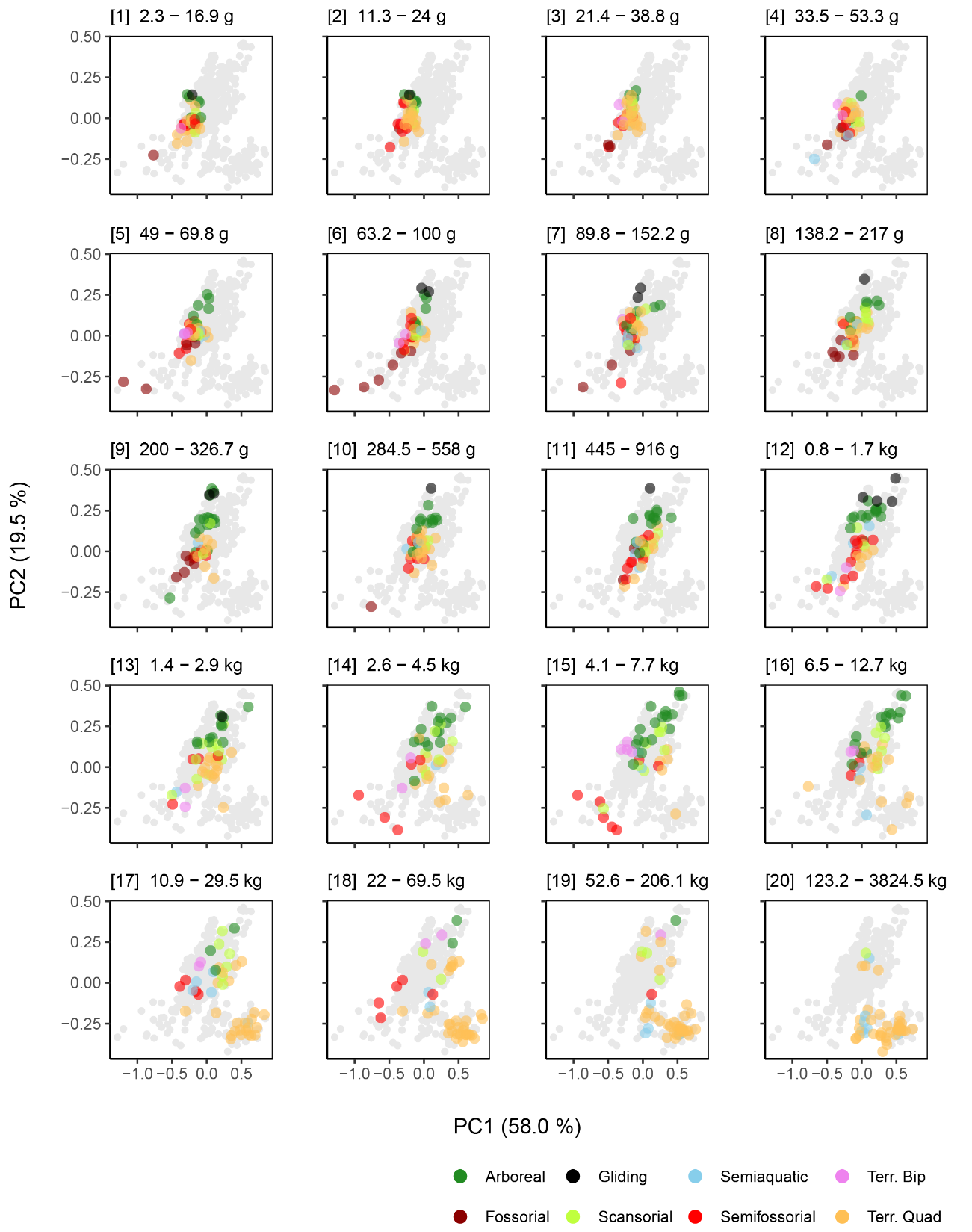


**Figure S6. Forelimb morphospace across body mass ranges.** Forelimb PC1 and PC2 morphospace of terrestrial mammals, indicating the species belonging to 20 different body mass ranges (overlapping mass bins). Species included at each bin are colored according to their locomotor ecology, while species that do not belong to the overlapping bin are grey. Body mass range of each rolling bin is specified on the top of each panel.


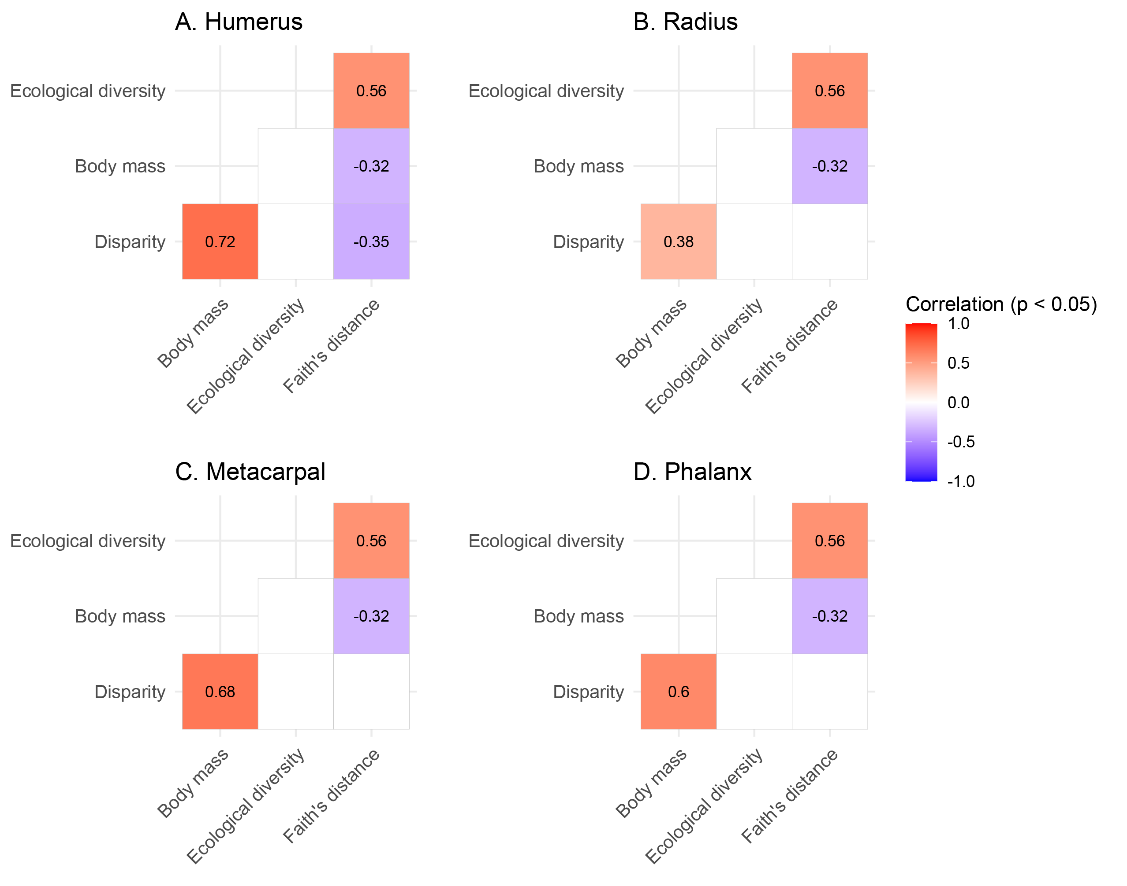


**Figure S7. Correlation between bone shape disparity, body mass, ecological diversity and Faith’s distance**. The heatmaps illustrate the results of significant values obtained from a Kendall correlation test, using all mass bins. A) Correlation for the humerus; B) Correlation for the radius; C) Correlation for the third metacarpal; D) Correlation for the first phalanx of digit III.


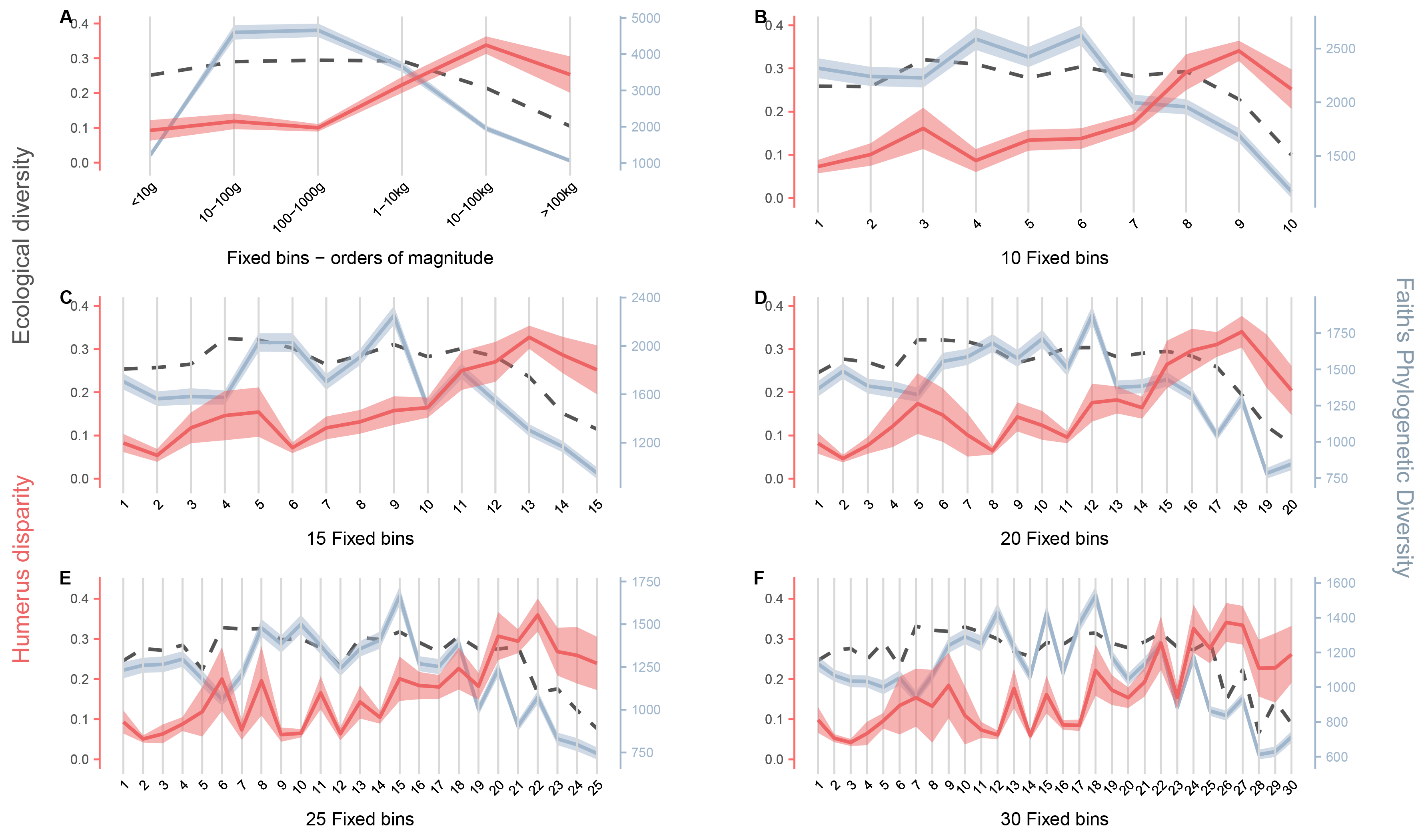


**Figure S8. Comparison of fixed binning methods on humerus shape disparity, ecological diversity and Faith’s phylogenetic diversity.** A) Species classified based on orders of magnitude; B) 10 fixed bins (66/67 species); C) 15 fixed bins (44/45 species per bin); D) 20 fixed bins (33/34 species per bin) *; E) 25 fixed bins (26/27 species per bin); F) 30 fixed bins (22/23 species per bin). * Binning number chosen to conduct downstream analyses. Disparity values are multiplied by 10 and Gower’s distance (Ecological diversity) is multiplied by 1.5 to facilitate plot visualization.


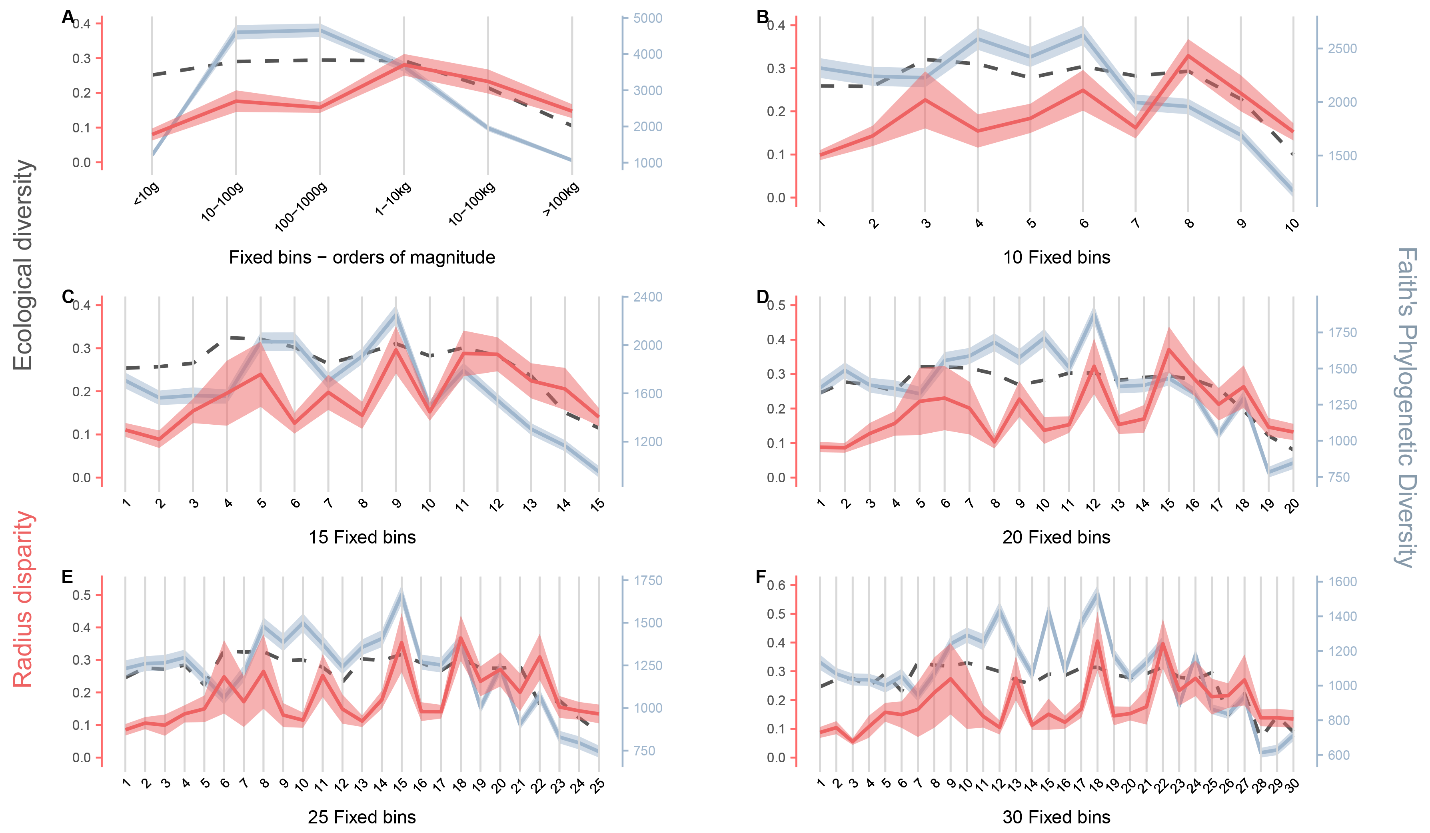


**Figure S9.** **Comparison of fixed binning methods on radius shape disparity, ecological diversity and Faith’s phylogenetic diversity.** A) Species classified based on orders of magnitude; B) 10 fixed bins (66/67 species); C) 15 fixed bins (44/45 species per bin); D) 20 fixed bins (33/34 species per bin) *; E) 25 fixed bins (26/27 species per bin); F) 30 fixed bins (22/23 species per bin). * Binning number chosen to conduct downstream analyses. Disparity values are multiplied by 10 and Gower’s distance (Ecological diversity) is multiplied by 1.5 to facilitate plot visualization.


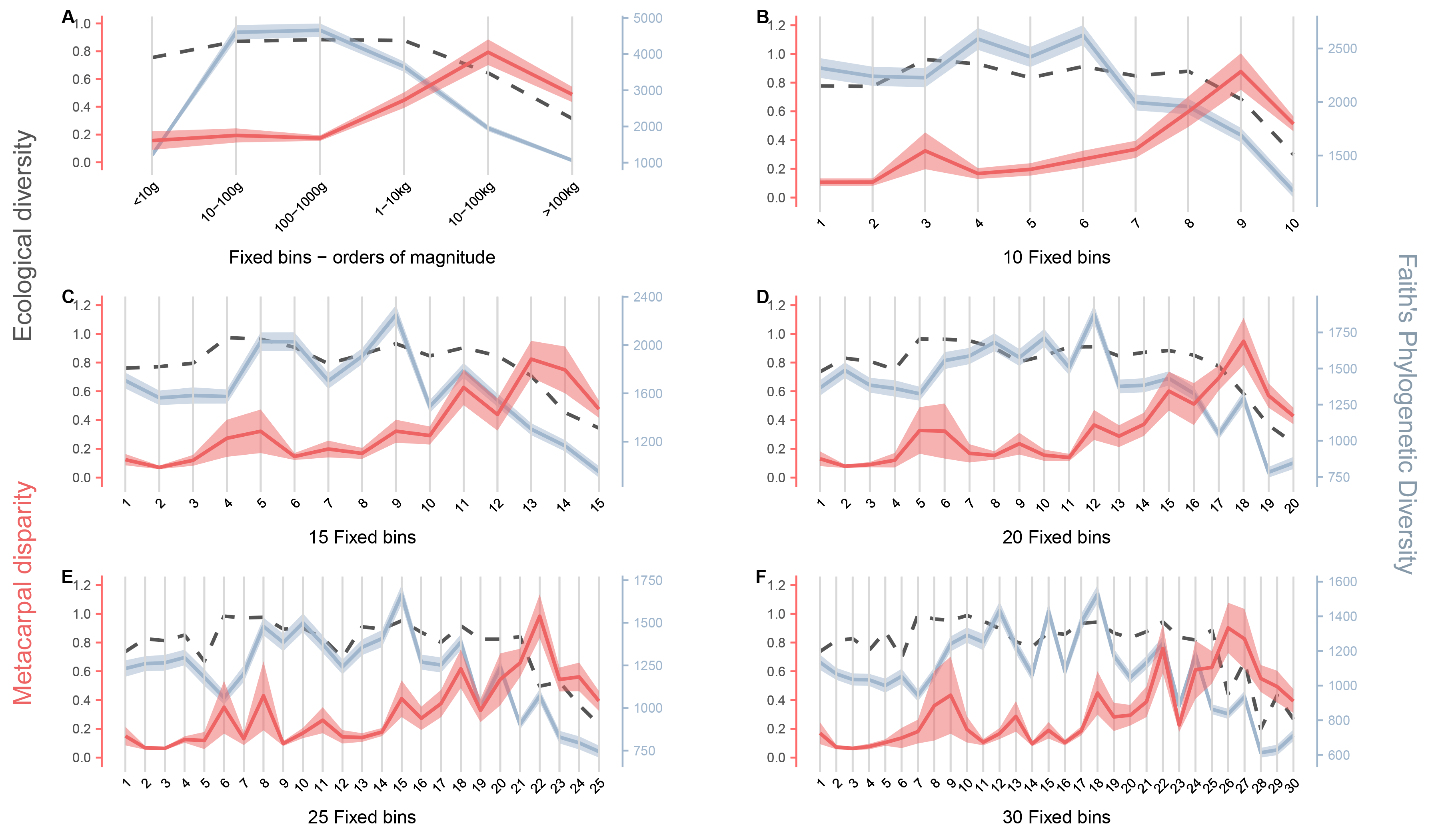


**Figure S10. Comparison of fixed binning methods on metacarpal shape disparity, ecological diversity and Faith’s phylogenetic diversity.** A) Species classified based on orders of magnitude; B) 10 fixed bins (66/67 species); C) 15 fixed bins (44/45 species per bin); D) 20 fixed bins (33/34 species per bin) *; E) 25 fixed bins (26/27 species per bin); F) 30 fixed bins (22/23 species per bin). * Binning number chosen to conduct downstream analyses. Disparity values are multiplied by 10 and Gower’s distance (Ecological diversity) is multiplied by 4.5 to facilitate plot visualization.


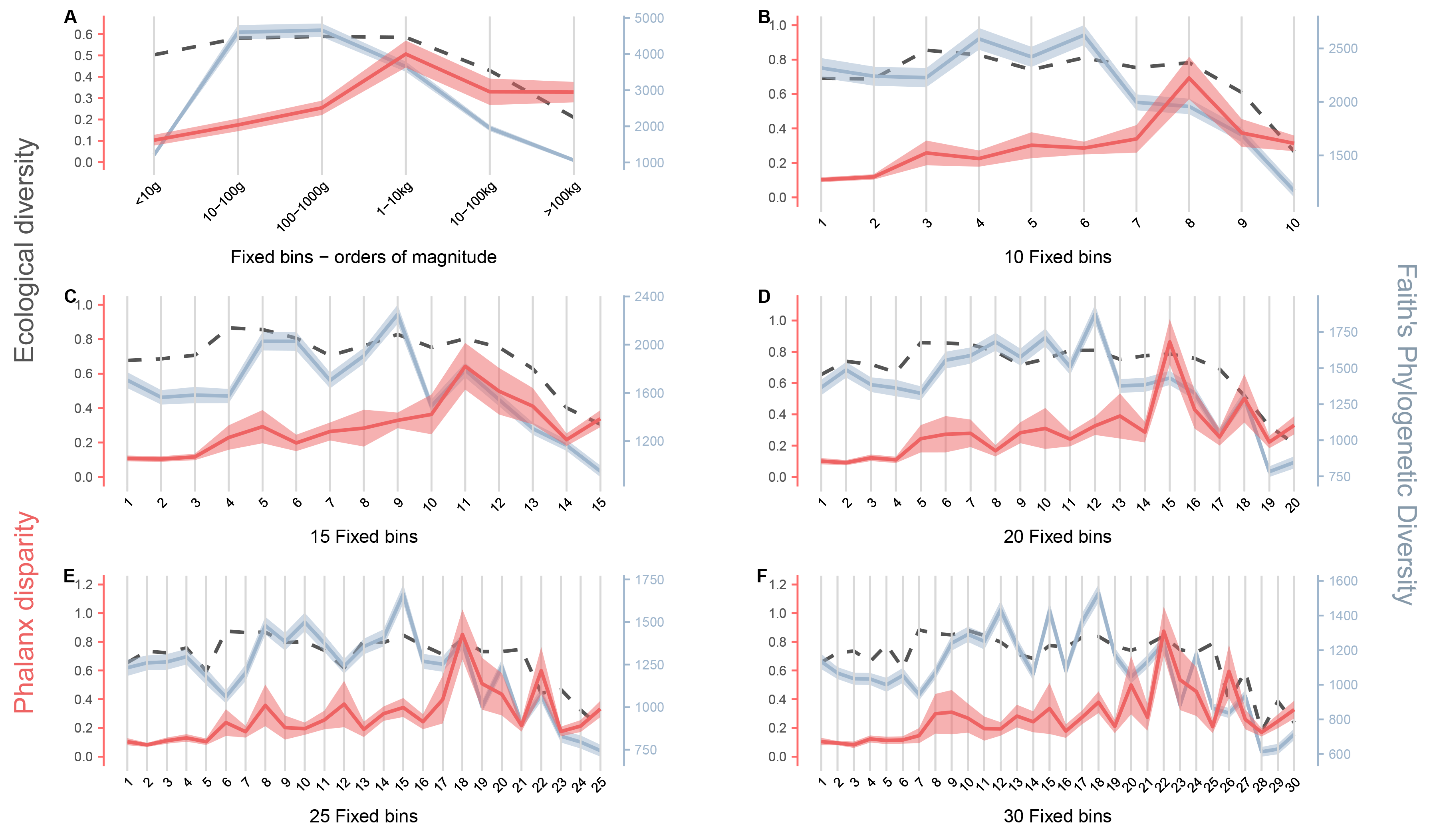
 **Figure S11.** **Comparison of fixed binning methods on phalanx shape disparity, ecological diversity and Faith’s phylogenetic diversity.** A) Species classified based on orders of magnitude; B) 10 fixed bins (66/67 species); C) 15 fixed bins (44/45 species per bin); D) 20 fixed bins (33/34 species per bin) *; E) 25 fixed bins (26/27 species per bin); F) 30 fixed bins (22/23 species per bin). * Binning number chosen to S4conduct downstream analyses. Disparity values are multiplied by 10 and Gower’s distance (Ecological diversity) is multiplied by 3 in A and by 4 in B-F to facilitate plot visualization.


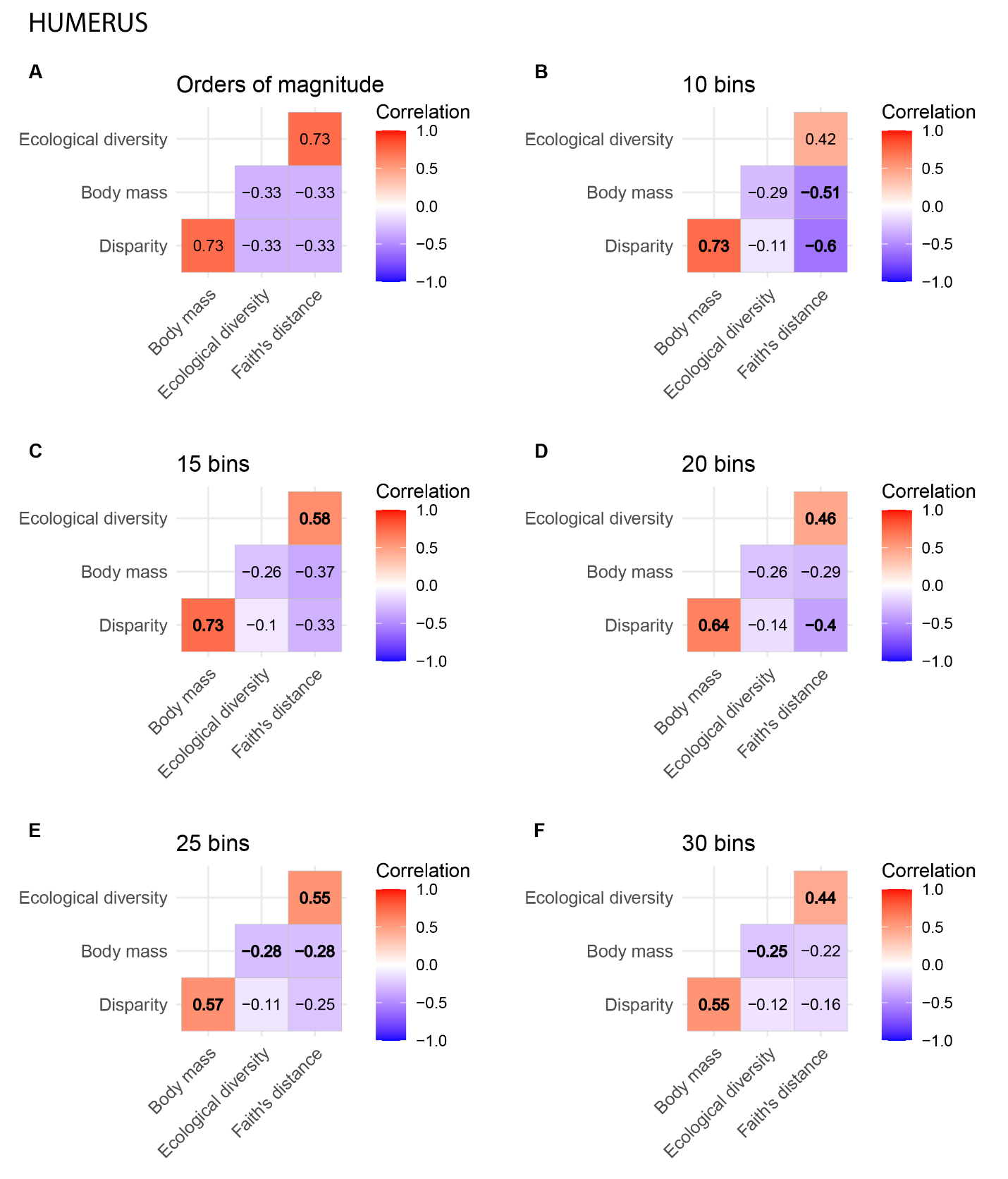


**Figure S12.** **Correlation between humerus shape disparity, body mass, ecological diversity and Faith’s distance with different fixed binning methods.** The heatmaps indicate the degree of correlation obtained from a Kendall correlation test. Values in bold are significant. A) Binning based on orders of magnitude; B) 10 fixed bins; C) 15 fixed bins; D) 20 fixed bins; E) 25 fixed bins; F) 30 fixed bins.


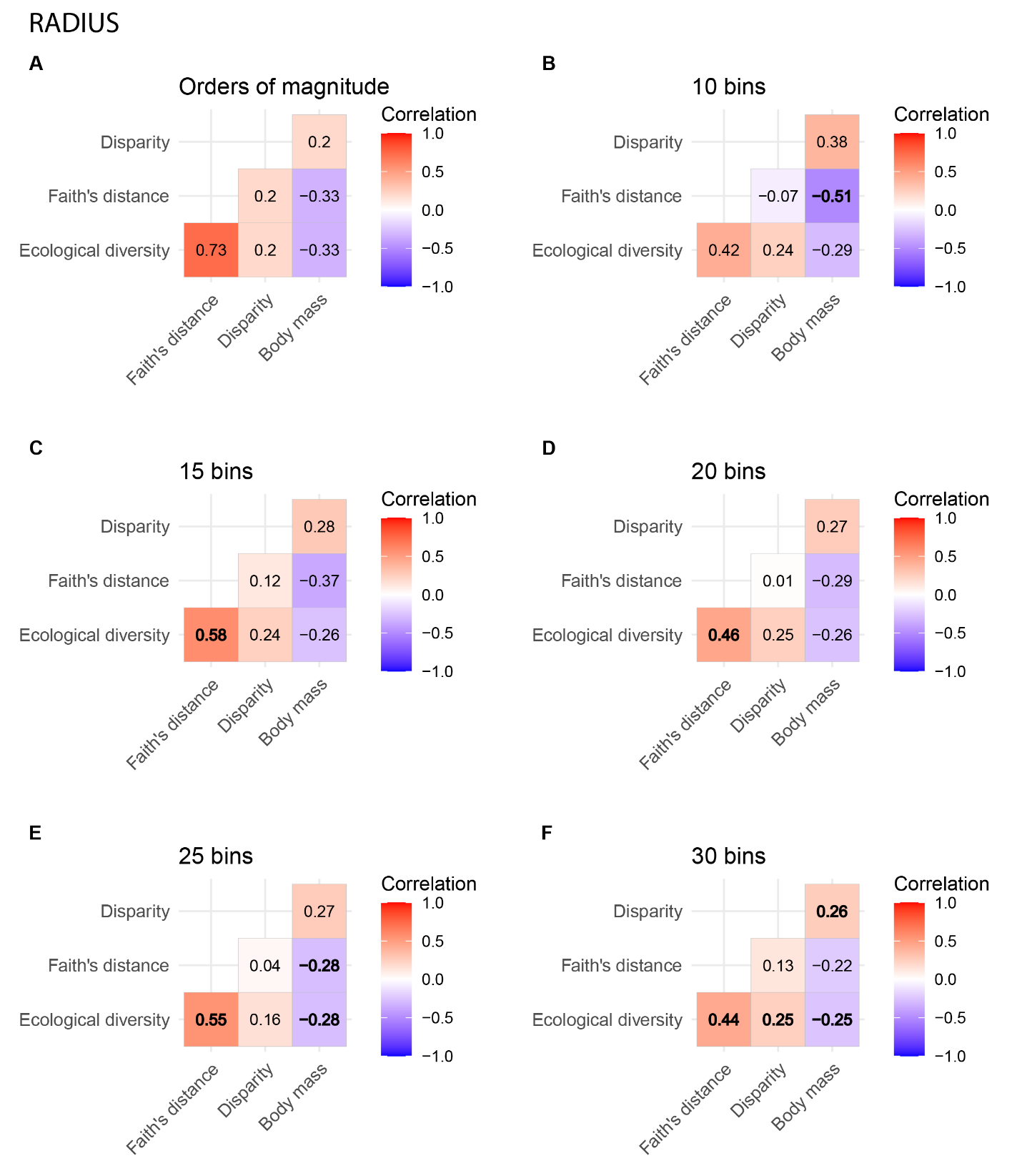


**Figure S13.** **Correlation between radius shape disparity, body mass, ecological diversity and Faith’s distance with different fixed binning methods.** The heatmaps indicate the degree of correlation obtained from a Kendall correlation test. Values in bold are significant. A) Binning based on orders of magnitude; B) 10 fixed bins; C) 15 fixed bins; D) 20 fixed bins; E) 25 fixed bins; F) 30 fixed bins.


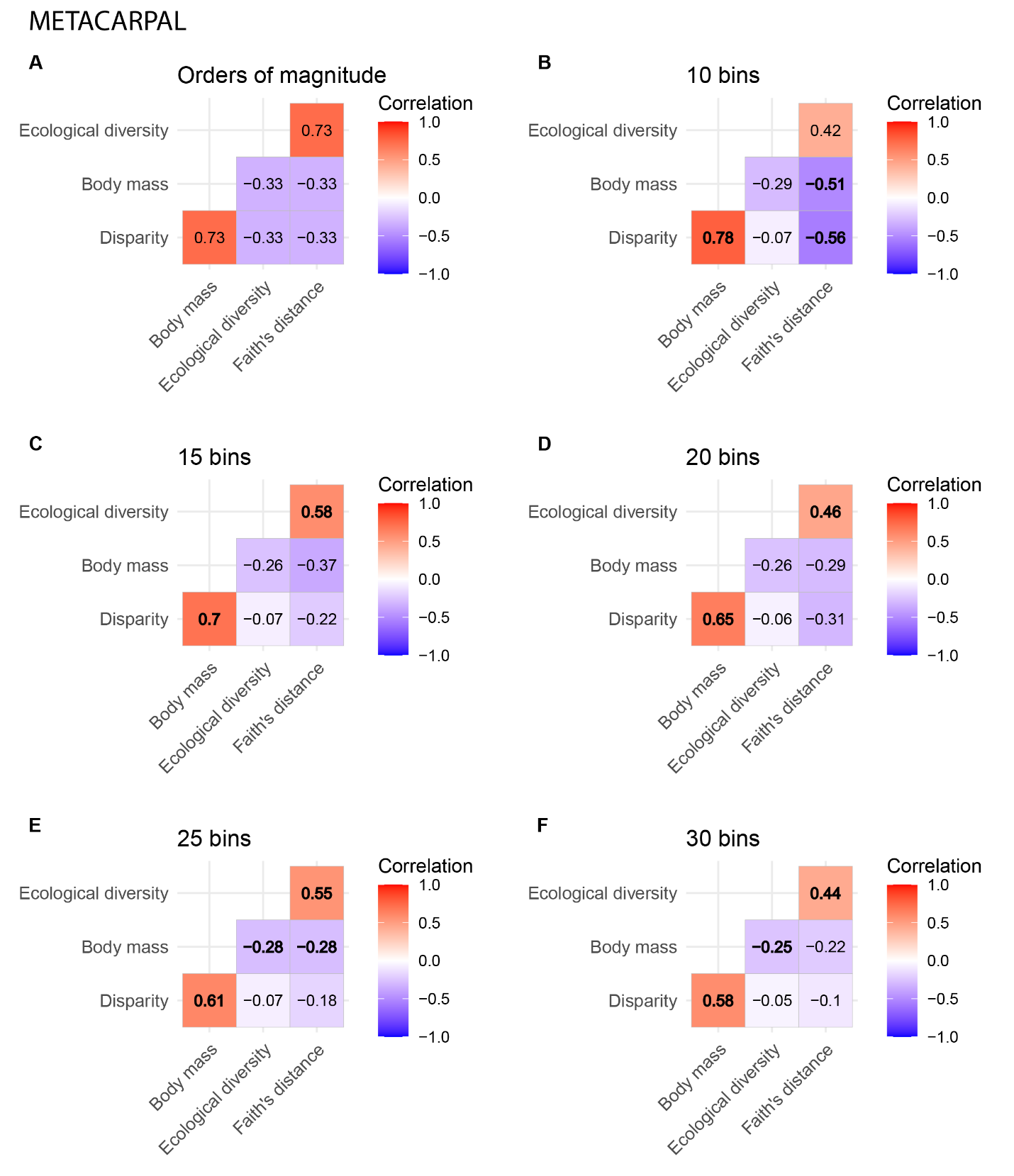


**Figure S14. Correlation between metacarpal shape disparity, body mass, ecological diversity and Faith’s distance with different fixed binning methods.** The heatmaps indicate the degree of correlation obtained from a Kendall correlation test. Values in bold are significant. A) Binning based on orders of magnitude; B) 10 fixed bins; C) 15 fixed bins; D) 20 fixed bins; E) 25 fixed bins; F) 30 fixed bins.


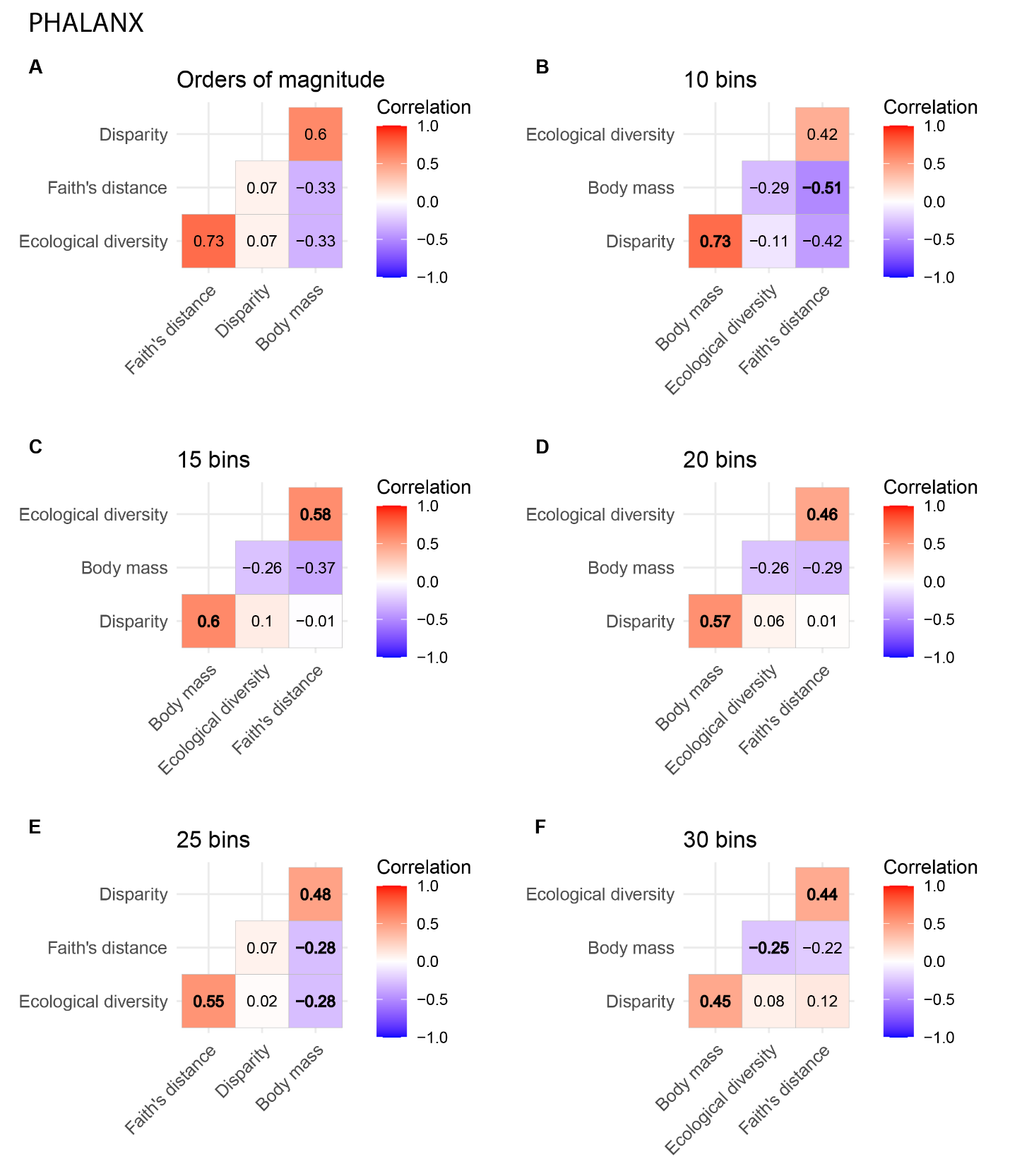


**Figure S15. Correlation between phalanx shape disparity, body mass, ecological diversity and Faith’s distance with different fixed binning methods.** The heatmaps indicate the degree of correlation obtained from a Kendall correlation test. Values in bold are significant. A) Binning based on orders of magnitude; B) 10 fixed bins; C) 15 fixed bins; D) 20 fixed bins; E) 25 fixed bins; F) 30 fixed bins.
